# Commentary on Garas et al., “Longitudinal Suicide Risk in Children and Adolescents With Attention Deficit and Hyperactivity Disorder: A Systematic Review and Meta‐Analysis”

**DOI:** 10.1002/brb3.71127

**Published:** 2026-03-10

**Authors:** Rachel E. Dew, Sudha R. Raman

**Affiliations:** ^1^ Department of Psychiatry and Behavioral Sciences Duke University School of Medicine Durham North Carolina USA; ^2^ Department of Population Health Sciences Duke University School of Medicine Durham North Carolina USA

1

To the Editor:

A recent systematic review by Garas et al. (https://onlinelibrary.wiley.com/doi/10.1002/brb3.70618; “Longitudinal Suicide Risk in Children and Adolescents With Attention Deficit and Hyperactivity Disorder: A Systematic Review and Meta‐Analysis”) delves into an important topic with implications for all domains of clinical care, as well as public health efforts. In updating their previous systematic review, with expansion to include meta‐analysis, the authors use rigorous methodology to track connections over time between clinical ADHD diagnosis in youth and later suicidal thoughts or behaviors, using cohorts followed up to 27 years.

As ADHD researchers and clinicians, we are increasingly struck by the need to develop behavioral science research around self‐harm and ADHD. Until recently, most scholarship on suicide risk conveyed by mental disorders failed to consider ADHD at all. Recent systematic reviews on the topic discuss risk conveyed by major depression, schizophrenia, substance use disorders, and borderline personality disorder (Favril et al. 2022; Knipe et al. 2019; Pigoni et al. 2024).

Suicidal phenomena in those with ADHD are poorly understood. They are also increasingly relevant, as the rate of pre‐pubertal children presenting to emergency rooms with suicidal thoughts and behaviors increase, with the most prevalent mental disorder in this young group being ADHD (Ben‐Yehuda et al. 2012). We applaud these authors’ efforts and would like to add some additional considerations for this field of inquiry.

Anecdotally, we find that it is rare for healthcare providers to fully appreciate a connection between suicidality and ADHD. Health professionals have typically been taught that ADHD falls into the classification of “externalizing disorders,” characterized by the effects the symptoms have on people around the patient. Other diagnoses associated with the term “externalizing” include oppositional defiant disorder and conduct disorder. These syndromes are literally defined by the behaviors that create problems for others and not by the internal experience of the patients themselves; in contrast, diagnostic criteria for “internalizing” disorders, such as depression and anxiety, give more weight to how the patients subjectively experience the world. If patients carry these latter diagnoses, providers know they are suffering.

In reality, ADHD patients are not usually oblivious to the impact of their symptoms, and this precipitates significant internalized stress. ADHD is a persistent, lifelong personal burden—a constant source of shame, failure, and demoralization. Many children and adolescents with ADHD come to expect that every day they will arrive late for school, forget their homework, be scolded for hyperactive behavior, annoy other students, accidentally offend people, and lose their temper. Over time, the constant refrains of “What is wrong with you?,” “Why would you do that?,” and “Everyone else can do this, why can't you?” lead to an answering call of “I give up.” Even if treatment is started, motivation becomes permanently diminished. These problems are self‐propagating; with more failures, those with ADHD start to avoid challenges, leading to even more failures.

Garas et al. expose critical gaps in our current understanding of a significant problem, pointing out that this research will be most valuable if mediating pathways can be determined. The paper provides an excellent discussion of proposed mediators and moderators, with varying levels of empirical support. These include gender; comorbid diagnoses; ADHD subtypes and severity; and common problematic experiences like transition to adulthood and family conflicts. Unfortunately, the available literature does not allow for a quantitative analysis of mediation in this review, and a qualitative review of the available data yields mixed findings.

While ADHD is a risk factor for many comorbid psychopathologies, we recommend serious consideration of a direct effect. We propose that in a large proportion of cases there is no intermediate diagnosis—that ADHD is itself a strong, independent risk factor for suicidal thoughts and behaviors (Austgulen et al. 2023).

For most patients, ADHD affects all areas of functioning. It is possible that both ADHD symptoms and their consequent social effects serve as partial mediators in this complex relationship, and these factors may operate independent of comorbid syndromes, such as depressive or anxiety disorders. This may be more apparent in certain subpopulations, for example, young children. Although Garas et al. found significant effects of ADHD on suicidality only in longer cohorts, it has been observed that suicidal thoughts in children as young as 10 are more likely to occur in ADHD. Prior to puberty, both major depressive disorder and substance use disorders are rare, yet in this group, ADHD remains a clear risk factor for suicidality; this finding weakens the argument for mediation through both extended time and comorbid conditions (Ben‐Yehuda et al. 2012; R. T. Liu et al. 2022; Marraccini et al. 2021).

Another indicator that ADHD may be a specific and independent risk factor for suicidal behavior is the robust emerging evidence, touched on by Garas et al., that stimulant medication treatment for ADHD prevents suicidal thoughts and behaviors (L. Li et al. 2025; W. Liu et al. 2020; Man et al. 2017; Siffel et al. 2020; Zhang et al. 2025). While further exploration of the precise mechanisms underlying this finding is necessary, this makes complete mediation through comorbidities unlikely.

If proven, this protective stimulant effect is at least as impressive as that documented with antidepressants (Huang et al. 2022; K. Li et al. 2022). One reason to appreciate the possible direct from ADHD to self‐harm is to guide treatment. If it is assumed that a mood disorder must be involved, this may become main pharmacologic target, possibly missing an opportunity for the protective effect of stimulant medications.

In summary, suicidal thoughts and behaviors in ADHD patients are important clinical phenomena that significantly impact healthcare across settings. Currently there are no specific treatments for self‐harming thoughts and behaviors in youth with ADHD. It is unknown if approaches designed for other groups, which largely depend on cognitive flexibility and advanced planning, are effective here. Thus, it is hoped that this review will spur more of this type of research, critical in the development of tailored prevention and treatment strategies for this vulnerable group.

## Conflicts of Interest

The authors declare no conflicts of interest.
